# Pulsed electromagnetic field ameliorates the progression of osteoarthritis via the Sirt1/NF-κB pathway

**DOI:** 10.1186/s13075-025-03492-0

**Published:** 2025-02-14

**Authors:** Siqi Zhou, Haiyan Wen, Xiongwei He, Xiaotao Han, Haohuan Li

**Affiliations:** 1 Department of Orthopedics, Renmin Hospital of Wuhan University, Wuhan, 430060 China; 2 Department of Pharmacy, Renmin Hospital of Wuhan University, Wuhan, 430060 China; 3 Wuhan National High Magnetic Field Center, Huazhong University of Science and Technology, Wuhan, 430074 China; 4 State Key Laboratory of Advanced Electromagnetic Engineering and Technology, Huazhong University of Science and Technology, Wuhan, 430074 China

**Keywords:** PEMF, IL-1β, Inflammation, Sirt1/NF-κB signaling pathway, Osteoarthritis

## Abstract

**Background:**

Pulsed electromagnetic field (PEMF) is a non-invasive treatment that utilizes electromagnetic fields to reduce inflammation and promote tissue repair. However, PEMFs’ anti-inflammatory effect on osteoarthritis (OA) and the potential mechanism has not been fully elucidated.

**Methods:**

Human chondrocytes (C28/I2) were stimulated with interleukin (IL)-1β with or without the treatment of PEMF. CCK-8 assay Kit was used to detect cell viability. RT-qPCR, ELISA, immunofluorescent staining and western blot was used to analyze relative markers of inflammatory response and extracellular matrix (ECM) under the treatment of PEMF and related mechanism. Besides, the significance role of Sirt1 was assessed by using the Sirt1 inhibitor (EX-527). Moreover, immunohistochemistry and immunofluorescence staining were carried out to evaluate the curative effect of PEMF on OA mice induced by the destabilization of the medial meniscus (DMM).

**Results:**

PEMF inhibited IL-1β-mediated the expression of pro-inflammatory factors. Besides, PEMF alleviated IL-1β-induced degradation of ECM by increasing the expression of Col2a1 and ACAN, while inhibiting the expression of MMP13 and ADAMTS5. At the mechanism level, PEMF increased the expression of Sirt1 and inhibited IL-1β-induced the activation of NF-κB pathway. Furthermore, blocking Sirt1 with EX-527 attenuated the effect of PEMF on the inhibition of NF-κB pathway and the expression of ECM in IL-1β-induced chondrocytes. In vivo, PEMF-treated OA mice showed low modified mankin scores, reduced the number of osteophytes and preserved joint structure.

**Conclusions:**

Our results suggest that PEMF inhibits NF-κB pathway and blocks the expression of inflammatory factors by activating the expression of Sirt1, which may be a novel strategy for OA.

**Supplementary Information:**

The online version contains supplementary material available at 10.1186/s13075-025-03492-0.

## Introduction

Osteoarthritis (OA) is a chronic and degenerative disease characterized by the loss of cartilage, osteophyte formation and alterations in subchondral bone [1, 2]. As the leading cause of disability, OA has affected approximately 240 million people globally, particularly the elderly, bringing an increased strain on health-care systems [3]. Currently, the nonsteroidal anti-inflammatory drugs (NSAIDs) is common non-surgical treatment used in OA patients to reduce pain temporarily despite their side effect and drug interaction, whereas joint replacement surgery is recommended as an optimal therapy for patients in end-stage OA [4, 5]. Thus, an effective and safe intervention that can both control pain and improve joint function is in urgent demand, especially for those in early-stage OA.

Inflammation plays a critical role in OA progression as it involves in the whole pathological process of OA by releasing pro-inflammatory factors to accelerate joint deterioration [6]. There are multiple pro-inflammatory cytokines in OA, among which the interleukin-1β (IL-1β) is a master regulator of pro-inflammation that mediates the expression of numerous genes related to secondary inflammation response [7, 8]. Moreover, growing evidence suggested that IL-1β contributed to the imbalance between the synthesis and degradation of extracellular matrix (ECM), leading to cartilage destruction in OA [9]. Studies had also shown that a high IL-1β level was found in synovial membrane and serum of OA patients, suggesting IL-1β overexpression was a causative factor of OA [10, 11]. Therefore, blocking IL-1β is regarded as a promising therapeutic strategy for treating OA [12].

Pulsed electromagnetic fields (PEMF) is a promising medical technology applied in tissue repair and regeneration and arise increasing attention owing to its advantage of effectiveness and non-invasiveness [13]. Studies have shown that PEMF can promote cartilage regeneration and repair [14]. Additionally, additional research has demonstrated that PEMF can reduce the expression of pro-inflammatory cytokines [15, 16]. As a consequence, we propose a hypothesis that PEMF might repair the damaged articular cartilage and inhibit inflammation to exert protective effect on OA.

In this study, we aimed to confirm the therapeutic effect of PEMF on OA and explored its underlying mechanism. Both in vitro and in vivo experiments were employed to investigate the effect of PEMF on inflammatory response, ECM degradation, and pathological changes of joint. These findings will provide novel evidence that PEMF is a promising therapy to improve joint function of OA patients.

## Materials and methods

### Chemicals and reagents

Recombinant human IL-1β was sourced from MedChemExpress (NJ, USA). TRIzol reagent was acquired from Thermo Fisher Scientific. (CA, USA). 2×ChamQ Universal SYBR qPCR Master Mix and HiScript III RT SuperMix for qPCR (+ gDNA wiper) was obtained from Vazyme Biotechnology Co., Ltd. (Nanjing, China). Primary antibodies against iNOS, Cox-2, Col2a1, and IκBα were acquired from Affinity Biosciences (Jiangsu, China). Primary antibody of anti-MMP13 was provided by ABclonal Technology Co.,Ltd.(Wuhan, China). Primary antibody of anti-P65 and anti-P-P65 were provided by Proteintech (Wuhan, China). FITC-conjugated Goat anti-Rabbit IgG (H + L) secondary antibody was also obtained from ABclonal Technology Co., Ltd. (Wuhan, China). Fetal Bovine Serum was purchased from Dcell biologics (Shanghai, China). DMEM/F12 medium and 0.25% trypsin was obtained by Gibco (NY, USA).

### Cell viability assays

Using the Cell Counting Kit-8 (CCK-8) assay to measure cell viability. Briefly, Human chondrocytes(C28/I2) were added in 96-well plates and induced by 10 ng/mL IL-1β for 24 h alone or simultaneously exposed to PEMF (75 Hz, 1.5mT) for 24 h. Subsequently, after adding 10 µl CCK-8 solution according to the instructions, the cell viability was measured by fluorescence micropore apparatus (Olympus, Japan).

### ELISA

Chondrocytes were seeded in 6-well plates and intervened according to the above description, the concentration of NO in cell culture supernatant was quantified by Griess reagent (Beyotime, Shanghai, China). According to the manufacturer’s instructions, using ELISA kits (R&D Systems, Minneapolis, MN, United States) to determine the levels of other inflammatory factors in the culture supernatant.

### Western blotting

RIPA lysis buffer (Beyotime, Shanghai, China) and BCA assay kit (Beyotime, Shanghai, China) were respectively utilized to isolate and detect the protein concentration. After isolating by using SDS-PAGE (10–12.5%), the samples were transferred to polyvinylidene fluoride (PVDF) membranes. Then, the membranes were blocked with 5% skim milk and incubated with primary antibodies at 4 ℃ overnight. Finally, after incubating with secondary antibodies, the images were captured by chemiluminescence Image analysis system (Tanon, Shanghai, China) and analyzed by Image J software.

### Immunofluorescence staining

Cells were added in 6-well plates and intervened according to the above description. The cells were fixed with 4% paraformaldehyde for 15 min. Then, permeated with 0.1–0.5% Triton X-100 for 10 min. Subsequently, after blocking with 3% bovine serum albumin (BSA) for 30 min, the cells were incubating primary antibodies at 4 °C overnight. After incubating with fluorescein-conjugated secondary antibodies at room temperature for 1 h, the cells were stained with DAPI and examined using an inverted fluorescence microscope (Olympus, Japan).

### Real-time qPCR (RT-qPCR)

The total RNA was extracted through TRIzol reagent. The HiScript III RT SuperMix for qPCR (+ gDNA wiper) kit was used to reverse transcription of RNA to cDNA. qPCR was carried out by using 2×ChamQ Universal SYBR qPCR Master Mix. According to the Ct value, the expression of relative mRNA was calculated through 2^-ΔΔCT^. The GAPDH was used as reference gene. The primer sequences were presented in Table 1.

**Table 1:** Primer sequences for RT-qPCR

Genes	Forward Primer	Reverse Primer
Col2a1	GGATGGCTGCACGAAACATACCGG	CAAGAAGCAGACCGGCCCTATG
ACAN	AGTCACACCTGAGCAGCATC	AGTTCTCAAATTGCATGGGGTGTC
MMP13	ATGCAGTCTTTCTTCGGCTTAG	ATGCCATCGTGAAGTCTGGT
ADAMTS5	GAACATCGACCAACTCTACTCCG	CAATGCCCACCGAACCATCT
GAPDH	GACAGTCAGCCGCATCTTCT	GCGCCCAATACGACCAAATC

### Animal studies

Thirty C57BL/6 mice aged about 7–8 weeks were acquired from the Hubei Medical Scientific Academy Experimental Center. All animal studies have been approved by the Ethics Committee of Renmin hospital of Wuhan University. Mice were housed in a normal environment (indoor temperature 20–23 °C, 12-hour cycle of light and shade, humidity 50–60%). The mice were randomly divided into 3 groups (*n* = 10 per group): sham group, destabilization of the medial meniscus (DMM)-induced OA group and PEMF group (PEMF group: PEMF, 75 Hz, 1.5mT, 4 h once a day for 8 weeks). For DMM-induced OA group, exposing the right knee skin of the mice by removing the hair around the knee joint under general anesthesia. Then, cutting the knee skin to open the joint cavity by using a surgical blade. Subsequently, freeing muscles and ligaments on the side by using curved forceps. Finally, the medial meniscal ligament was transected with micro-surgical scissors. After treating under PEMF for 8 weeks, the joints of mice were harvested for subsequent experimental analyses.

### Hematoxylin-eosin and safranin O-fast green staining

Briefly, mice knee joints were fixed by immersing in 4% paraformaldehyde and decalcified in 10% EDTA for three weeks. Subsequently, the dehydrated specimens were embedded in paraffin, and sectioned by using a Leica microtome. Morphological analysis was performed by using hematoxylin-eosin (H&E) and safranin O-fast green (S-O) analysis. According to the Modified Mankin OA scoring system, each histological section was assigned a score based on the sum of cartilage structure (0–5), chondrocytes (0–3), Safranin-O staining (0–5) and tidemark (0–1) [17].

### Immunohistofluorescence staining

Immunohistofluorescence staining was used to assess the protein expression of Col2a1, MMP13 and Sirt1 in mice joints. Briefly, after performing the procedure of deparaffinization and ethanol gradient-based hydration, the sections were retrieved by citric acid buffer (PH6.0) microwave antigen retrieval. Then the sections were blocked with 3% BSA and incubated with primary antibodies at 4 °C overnight. After incubating with fluorescein-conjugated secondary antibodies for 1 h and staining with DAPI for 5 min, the images were captured by using an inverted fluorescence microscope.

### micro-CT analysis

The micro-CT system (µCT50, BRUKER Medical) was employed to analyze and visualize the cartilage structure in mice. Briefly, specimens were well placed in a transparent cylindrical groove (9 mm in diameter) with paper tape fixation to ensure that the longitudinal axis of the knees were perpendicular to the X-ray beam, with a spatial resolution of 5 μm (450 kV, 120 mA, 0.5 mm filter). After that, the resulting scanning profiles were further analyzed using CTvox 2.1 software to obtain 3D reconstruction images of knee joint.

### Statistical analysis

GraphPad Prism 8.0 software was used for performing data analysis. Quantitative data indicated as mean ± standard error of mean (SEM). For the comparison between two groups, Student’s t-test was employed. One-way analysis of variance (ANOVA) was used for determining statistical significance among multiple groups. Besides, for the analysis of Mankin scoring results, the Kruskal-Wallis H test was used. Statistically significant was considered when p-values less than 0.05.

## Results

### PEMF promoted the cell viability of IL-1β-treated chondrocytes

**Fig. 1:**
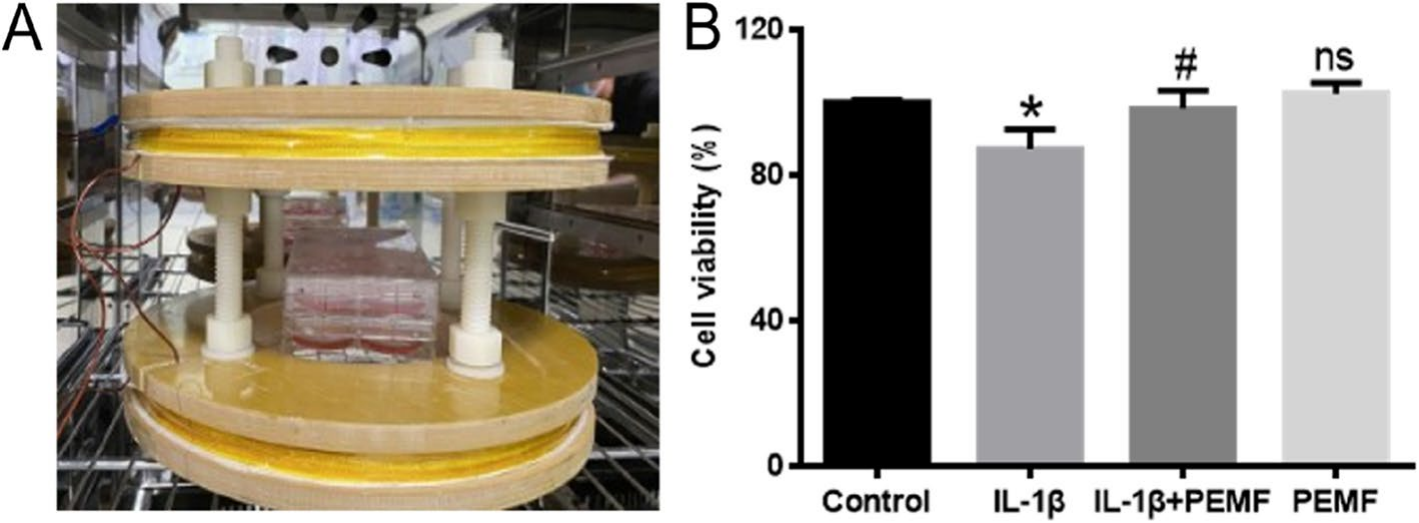
PEMF promoted the cell viability of IL-1β-treated chondrocytes. **A** Illustration of PEMF device. **B** The results of the cell viability detected by CCK-8 assy. * *p* < 0.05 vs. control group. # *p* < 0.05 vs. IL-1β group. ns, no significant difference

Figure 1A showed that chondrocytes were treated with PEMF in the CO_2_ incubator (Fig. 1A). As illustrated in Fig. 1B, IL-1β treatment significantly inhibited cell viability compared with the control group, while PEMF reversed these changes and promoted the cell viability of IL-1β-treated chondrocytes. Moreover, PEMF treatment alone had no significant cytotoxicity to chondrocytes.

### PEMF inhibited IL-1β-induced inflammatory response in chondrocytes

**Fig. 2:**
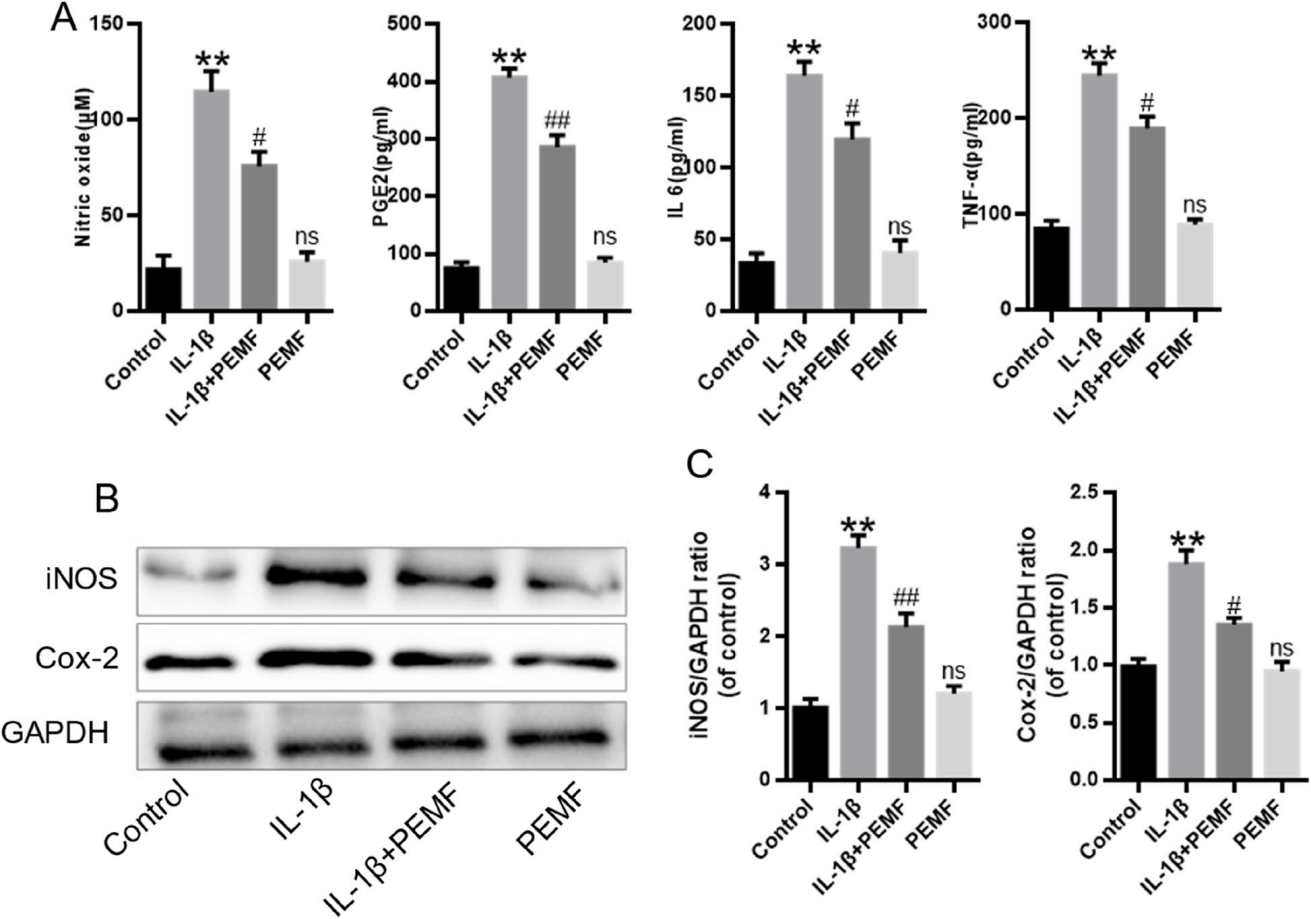
PEMF inhibited IL-1β-induced inflammatory response in chondrocytes. **A** The level of inflammatory factors in cell supernatant of chondrocytes. **B**, **C** The protein expression and quantification analysis of iNOS and Cox-2. * *p* < 0.05, ** *p* < 0.05 vs. control group. # *p* < 0.05, ## *p* < 0.01 vs. IL-1β group

IL-1β was used to induce an inflammatory response in chondrocytes, mimicking an arthritic condition in vitro [18]. In order to explore the impact of PEMF on inflammatory response in chondrocytes, we assessed the level of inflammatory mediators including nitric oxide (NO), prostaglandin E2 (PGE2), IL-6 and TNF-α by ELISA. Results showed that PEMF remarkably decreased IL-1β-stimulated the high level of NO, PGE2, IL-6 and TNF-α in cell supernatant of chondrocytes (Fig. 2A). The results of western blot analysis were consisted with these findings, showing reduced protein expressions of iNOS and Cox-2 in the IL-1β + PEMF group when compared with the IL-1β-treated group (Fig. 2B-C). These findings suggested that PEMF significantly inhibited IL-1β-induced inflammatory factors in chondrocytes.

### PEMF promoted the balance between matrix synthesis and degradation in IL-1β-induced chondrocytes

**Fig. 3:**
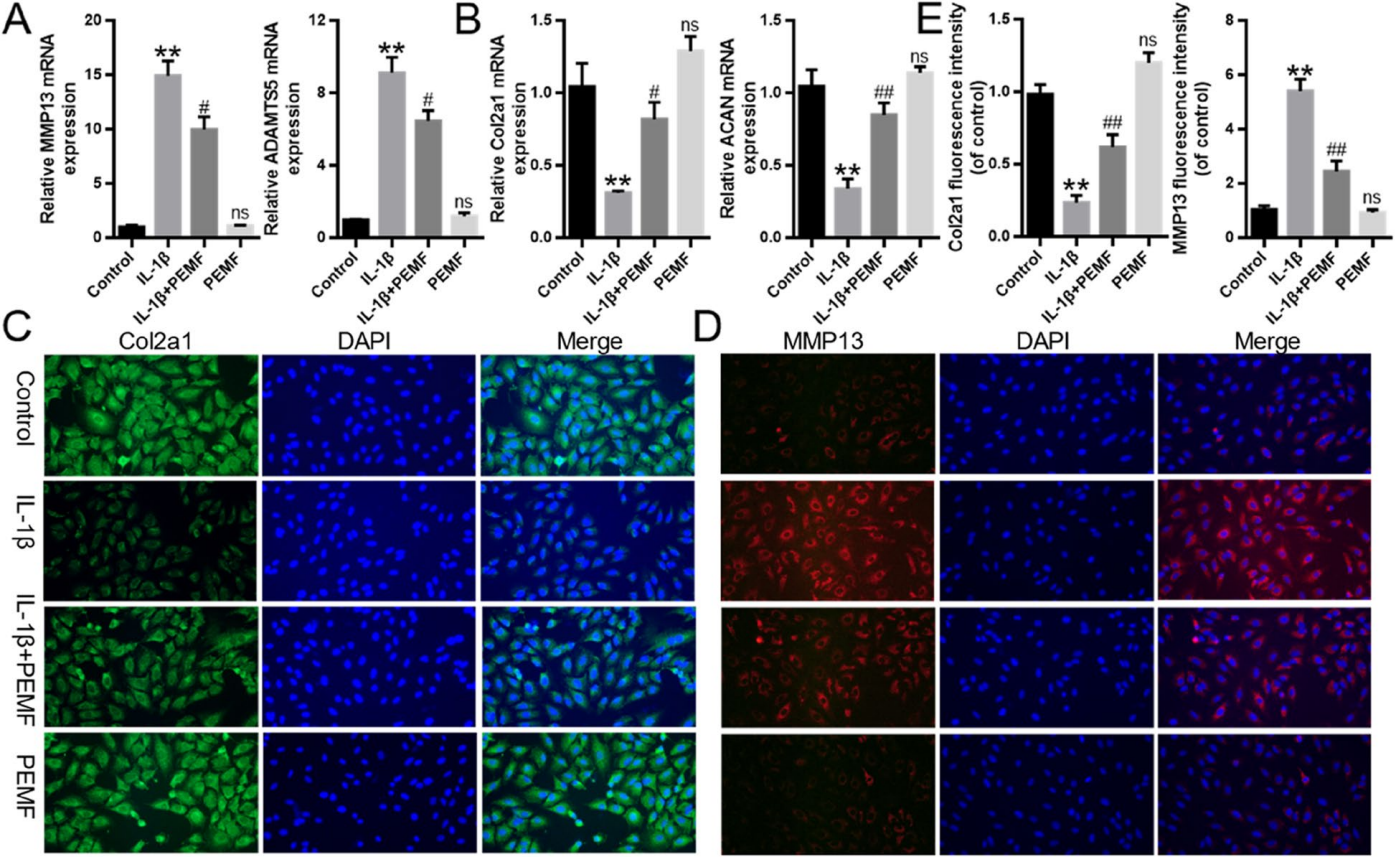
PEMF promoted the balance between matrix synthesis and degradation in IL-1β-induced chondrocytes. **A** and **B** RT-qPCR results. **C**-**E** Immunofluorescence was used to evaluate MMP13 and Col2a1 expression after the treatment of PEMF or IL-1β in chondrocytes (Scale bar:100 μm). * *p* < 0.05, ** *p* < 0.05 vs. control group. # *p* < 0.05, ## *p* < 0.01 vs. IL-1β group

IL-1β increases expression of MMPs, which leads to matrix degradation of chondrocytes [19]. As shown in Fig. 3A and B, compared with control group, IL-1β remarkedly induced the mRNA expression of MMP13 and ADAMTS5, and reduced the mRNA expression of Col2a1 and ACAN. However, compared with IL-1β group, the mRNA expression of MMP13 and ADAMTS5 in IL-1β + PEMF group significantly decreased, while the mRNA expression of Col2a1 and ACAN significantly increased. Immunofluorescence results demonstrated that PEMF effectively prevented matrix degradation by promoting Col2a1 expression but decreasing MMP13 expression when compared to that of IL-1β group (Fig. 3C-E) These findings suggested that PEMF treatment significantly promoted the balance between matrix synthesis and degradation in IL-1β-induced chondrocytes.

### PEMF suppressed the activation of NF-κB signaling

**Fig. 4:**
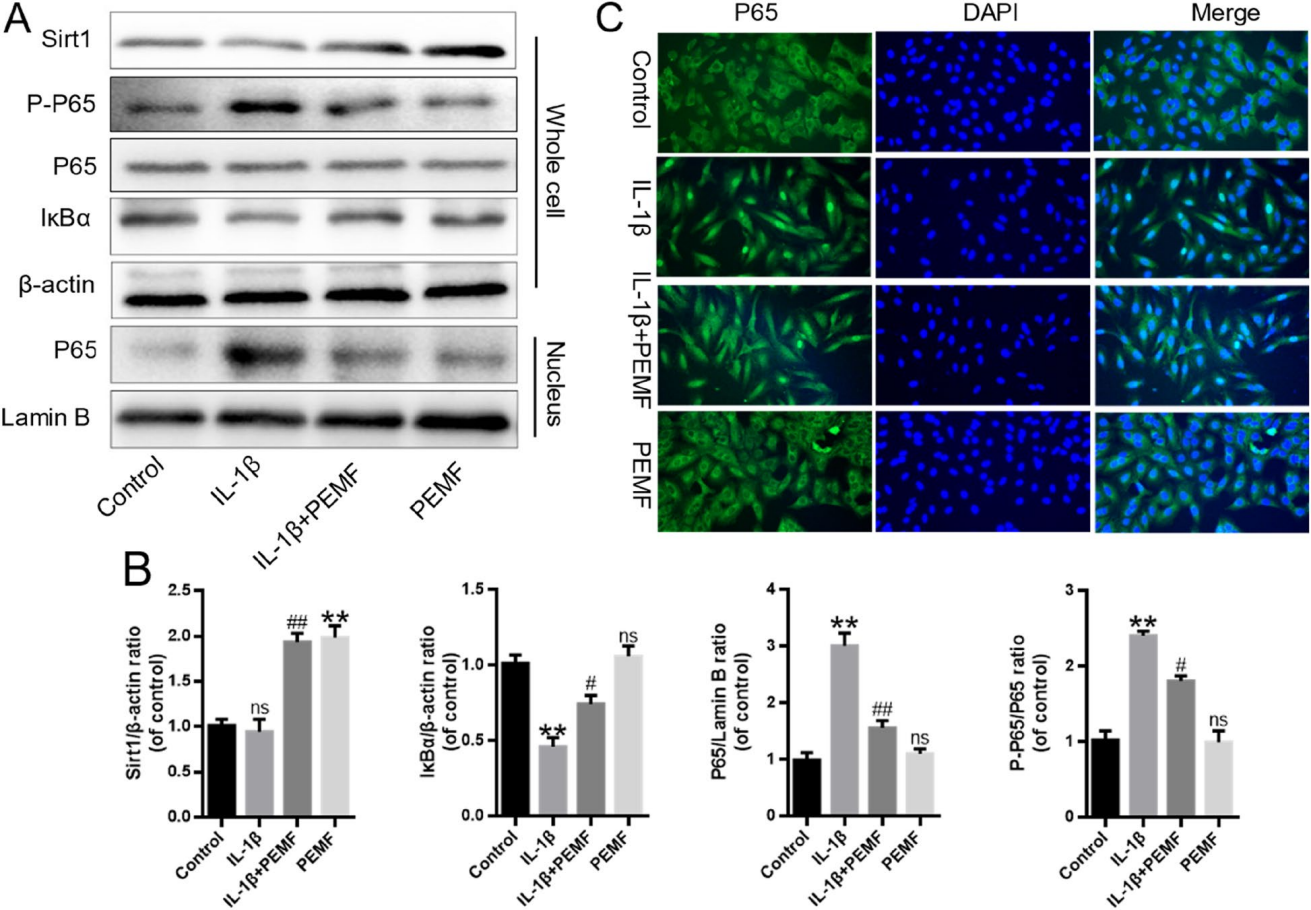
PEMF suppressed the activation of NF-κB signaling. **A**, **B** The Sirt1, P-P65, IκBα and P65 protein expression and statistical analysis in in IL-1β-induced chondrocytes. **C** Immunofluorescence was used to evaluate P65 expression after the treatment of PEMF or IL-1β in chondrocytes (Scale bar:100 μm). *p < 0.05, ** *p* < 0.01 vs. control group. # *p* < 0.05, ## *p* < 0.01 vs. IL-1β group

NF-kB is an essential component of the IL-1B-mediated inflammatory signaling pathway [20, 21]. To verify whether PEMF exerts its anti-inflammatory effects by inhibiting NF-kB, we conducted experiments using western blot and immunofluorescence. Based on the western blot findings, the ratio of P-P65/P65 protein in IL-1β-stimulated chondrocytes exhibited a significantly higher magnitude when compared to that of control group. PEMF treatment reduced the P-P65/P65 ratio significantly. Moreover, PEMF could reverse the increase of P65 expression and decrease of IκBα expression induced by IL-1β (Fig. 4A -B). These changes were also confirmed by immunofluorescence staining, which showed that PEMF inhibited the translocation of P65 to the nucleus stimulated by IL-1β, indicating that PEMF suppressed the activation of NF-κB signaling in IL-1β-induced chondrocytes (Fig. 4C).

### PEMF inhibited Sirt1-mediated the activation of NF-κB signaling

**Fig. 5:**
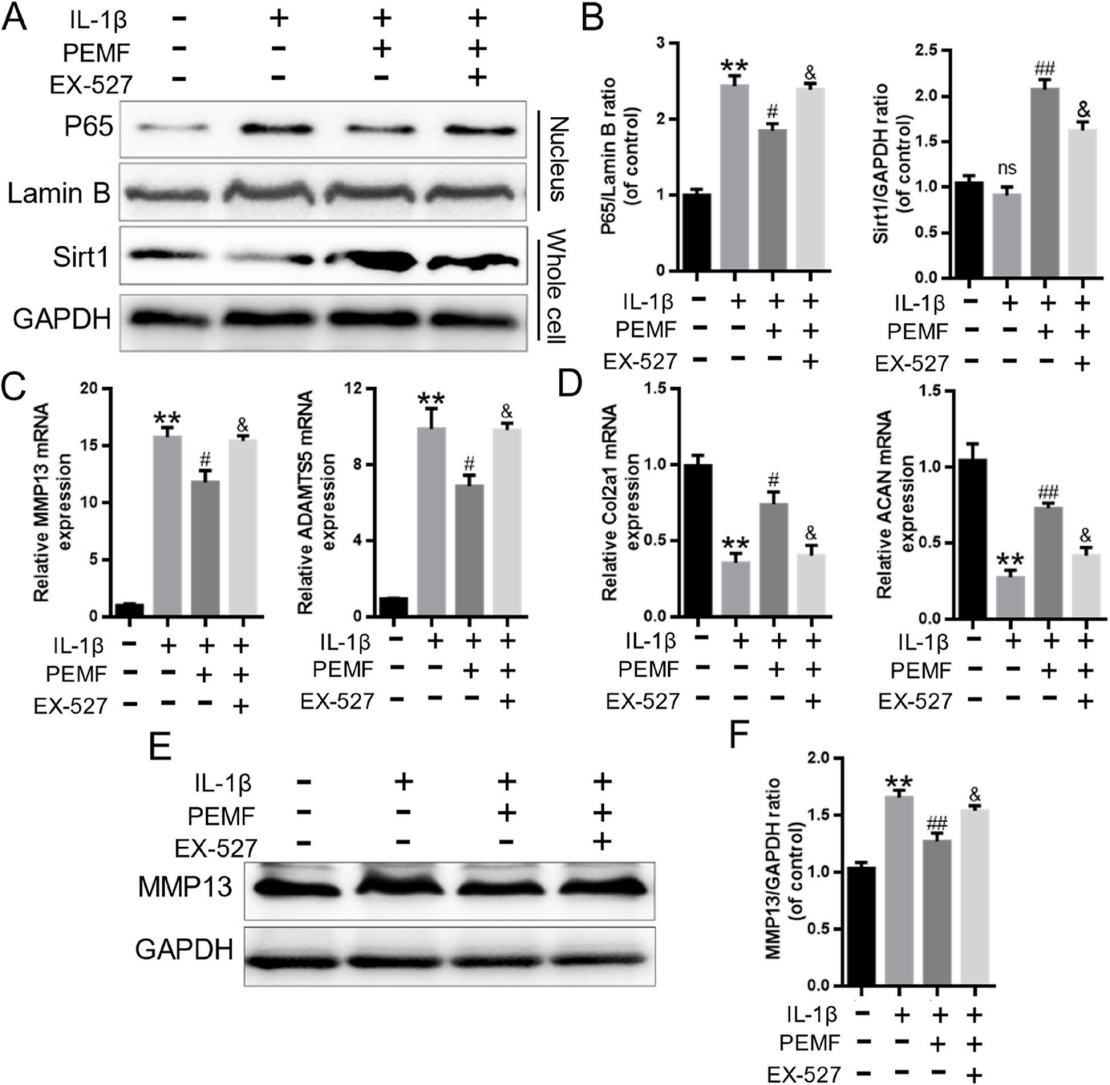
PEMF inhibited Sirt-mediated the activation of NF-κB signaling. **A**, **B** The Sirt1 and P65 protein expression and statistical analysis in chondrocytes. **C**, **D** RT-qPCR was used to detected the mRNA levels of MMP13, ADAMTS5, Col2a1 and ACAN treated with IL-1β and PEMF combined with or without Sirt1 inhibitor EX-527. **E**, **F** The MMP13 protein expression and statistical analysis in chondrocytes. * *p* < 0.05, ** *p* < 0.01 vs. control group. # *p* < 0.05, ## *p* < 0.01 vs. IL-1β group. & *p* < 0.05 vs. PEMF group

Sirt1 is a key regulator that modulates the inflammatory response [21]. The results of western blotting showed that compared with the control and IL-1β group, the expression of Sirt1 in the PEMF group was remarkedly elevated (Fig. 4A-B). The EX-527 is a well-established inhibitor of Sirt1. Moreover, to explore the key role of Sirt1 in the effect of PEMF on inhibition of NF-κB signaling, the EX-527 was used to block the effect of Sirt1. The results revealed that EX-527 effectively inhibited Sirt1 expression while increased the nuclear expression of p65 in chondrocytes (Fig. 5A-B). Besides, EX-527 also reversed the PEMF-induced the reduction in MMP13 and ADAMTS5 mRNA level, and increase in the Col2a1 and ACAN mRNA levels in chondrocytes (Fig. 5C-D). Furthermore, the results of western blotting showed that EX-527 could eliminate the low expression of MMP13 caused by PEMF (Fig. 5E-F). The above data suggested that PEMF inhibited NF-κB signaling pathway through the activation of Sirt1.

### PEMF alleviated OA progression in vivo

**Fig. 6:**
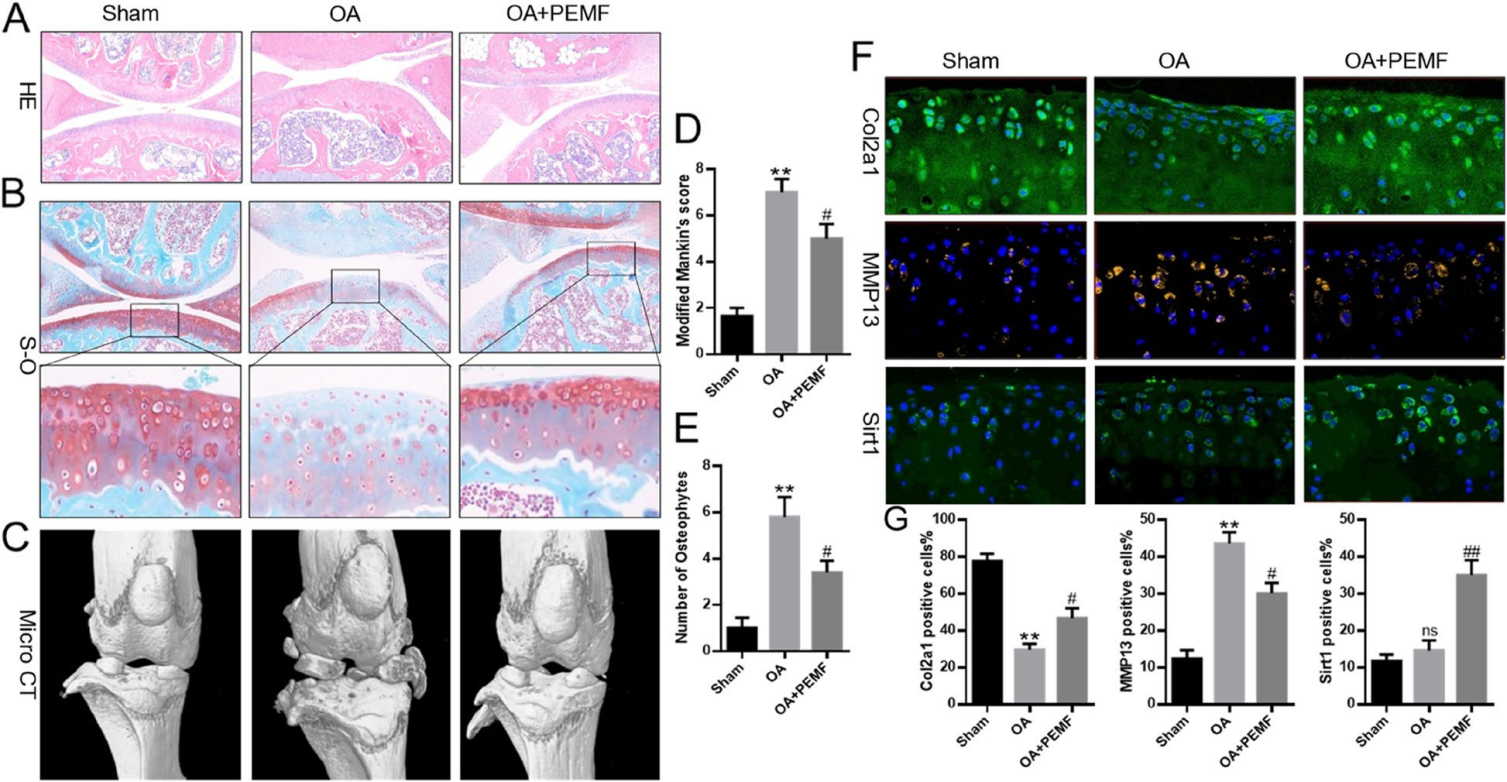
PEMF provided protection against OA progression in vivo. **A** and **B** H&E staining and Safranin-O staining (Scale bar:200 μm). **C** Representative micro-CT images of knee joint. **D** Modified Mankin scores of knee joint. **E** Number of osteophytes. **F** Immunofluorescence staining (Scale bar:20 μm). * *p* < 0.05, ** *p* < 0.01 vs. sham group. # *p* < 0.05, ## *p* < 0.01 vs. OA group

To explore the effect of PEMF in vivo, the mice OA models of DMM were established. The status of the mice knee joint cartilage was evaluated by H&E and S-O staining. As shown in Fig. 6A-B, compared with the OA group, PEMF treatment reduced the degree of deterioration of joint surface, the loss of proteoglycan and the erosion of joint. The results of modified mankin scoring system also revealed that, the OA + PEMF group had lower scores than OA group (Fig. 6D). Besides, the 3D structural alteration in OA mice was assessed by using micro-CT. The results showed that, in comparison to that of the sham group, the number of osteophytes in the OA group were remarkably increased. Conversely, PEMF treatment reduced the number of osteophytes induced by OA (Fig. 6C and E). The results of immunohistofluorescence staining indicated that the OA group exhibited reduced protein expression of Col2a1 and Sirt1, and overexpression of MMP13. PEMF treatment significantly reduced the expression of MMP13, and increased the expression of Col2a1 and Sirt1 (Fig. 6F and G). Above results indicated that PEMF slowed OA progression by attenuating cartilage degradation in OA mice.

## Discussion

The sustained cartilage damage and chronic inflammation are directly linked to the clinical symptoms of OA [19]. Therefore, the main goal of OA treatment is to repair the destruction of cartilage structure and control inflammatory response, but there is a long way for current non-surgical managements to achieve this goal. Despite the use of bisphosphonates have been reported to attenuate early OA in mice [22]. However, there are still limited selection for OA patient. The NASAIDs are recommended as the first-line treatment in both OARSI and ESCEO guidelines though their gastrointestinal, cardiovascular and renal side effects [20]. To explore an effective and safe strategy for OA treatment, we paid attention to the PEMF due to its property of non-invasiveness and good controllability, which might be a promising and effective approach in the management of OA symptoms.

Since the approval of PEMF therapy by the US Food and Drug Administration (FDA) for the treatment of nonunion fractures in 1979, growing studies about the promising application of PEMF in maintaining bone health have been reported [21, 22]. Studies have shown that PEMF therapy induces cellular responses in epidermal cells, fibroblasts, white blood cells, and nerve cells by enabling the generation of micro-currents and ion transport in living tissues, emerging as an alternative treatment for various inflammation-related diseases [23, 24]. For example, PEMF therapy can significantly reduce septic shock by decreasing the expression of proinflammatory cytokine genes [16]. Additionally, PEMF has demonstrated the ability to expedite cell differentiation, boost collagen deposition, and possibly restore vascular function to a state of homeostasis [25]. Its anti-inflammatory effect may be partially mediated by the membrane stabilization effect of PEMF, by restoring PMCA and intracellular Ca ^2+^ level to inhibit the biosynthesis of PGE2 [26]. Regardless, the effectiveness of PEMF depends on its amplitude, waveform as well as stimulation duration. Here, we revealed that the PEMF at 75 Hz, 1.5mT showed a protective effect on inflammation and matrix degradation in IL-1β-induced chondrocytes, as manifested by the decreased expression of inflammatory mediators (NO, PGE2, IL-6 and TNF-α) and genes that regulated matrix degradation (MMP13 and ADAMTS5) and the increased expression of genes that regulated matrix synthesis (Col2a1 and ACAN).

NF-κB is one of the important masters of inflammatory response, acting as a “rapid-acting” transcription factor to activate inflammation [27]. In normal physiological conditions, the p65 subunit of NF-κB protein interacts with the inhibitory protein NF-κB in the cytoplasm, leading to the formation of an inactive complex [28]. When cells stimulated by IL-1β, iκbα is phosphorylated and followed by degradation, which leads to the release and transfer of the P65 to the nucleus [29]. Within the nucleus, it can stimulate the NF-κB pathway and upgrade inflammation factors [30]. The upregulation of inflammatory factors such as iNOS leads to an increase of NO and MMPs, which leads to the degradation of the ECM and cartilage damage in OA [31]. This study showed that PEMF treatment significantly decreased nuclear expression of p65 and inhibited NF-κB activation induced by IL-1β.

Sirt1 is involved in the immune and inflammatory reaction cell functions such as the key factor adjustment [32]. According to Yeung et al. [33]’s study, it found that Sirt1 could inhibit the activation of NF-κb pathway by the inhibition of RelA/p65 subunits. Here, PEMF has been identified as an activator of Sirt1, which inhibited the down-stream NF-κB pathway. To explore the relationship between Sirt1 and NF-κB activation, Sirt1 inhibitors EX-527 was used to inhibit Sirt1 expression in chondrocytes. The results showed that PEMF suppressed the IL-1β-induced inflammation through the Sirt1-mediated NF-κB pathway in chondrocyte, which was reversed by the addition of EX-527. These findings suggested that PEMF reduced chondrocyte ECM degradation and inflammatory responses via the Sirt1-mediatd NF-κB signaling pathway.

Meanwhile, we also explored the effect of PEMF in DMM-induced OA mice. In the DMM mice, we observed the increased modified mankin scores and pathological changes including the fibrillation, fissures and erosion of cartilage and loss of matrix proteoglycan compared with the sham group. However, the PEMF treatment could effectively promote the cartilage metabolic homeostasis and improve the damaged cartilage, thereby alleviating OA phenotype in vivo. Thus, these findings in the present study clearly demonstrated the therapeutic effect of PEMF on anti-inflammation and cartilage repair, which might provide a safe and effective approach for OA treatment.

There are still several limitations. Firstly, we adopted one set of parameters for the application of PEMF in this study, and more various PEMF parameters will be used in our further studies to confirm the optimal parameters for OA treatment. Secondly, OA is a disease with complex pathological mechanisms, and we only explore the effect of PEMF from inflammation and matrix degradation but its effect on other process such as oxidative stress and ageing-related alterations are unclear. Moreover, the effect of PEMF needs to be verified in patients with OA in clinic, which will be performed in our future work.

## Conclusion

In summary, this study revealed the PEMF could effectively alleviate OA progression via improvement the IL-1β-stimulated inflammatory status and repair of cartilage destruction. Mechanically, PEMF inhibited NF-κB signaling pathway through the activation of Sirt1, but this effect of PEMF could be suppressed by the Sirt1 inhibitor EX-527. Our findings suggest the promising application of PEMF and provide a potential option for OA treatment.

### CRediT authorship contribution statement

S.q Zhou: conceptualization, methodology, validation, investigation, writing -original draft, funding acquisition. H.y Wen: Data Curation, methodology, formal analysis. X.w He: validation, investigation, data curation and writing -original draft. X.t Han: data curation, formal analysis, funding acquisition. H.h Li: writing -review & editing, supervision, funding acquisition.

## Supplementary Information

Supplementary Material 1

## Data Availability

No datasets were generated or analysed during the current study.
