# Supplementary material for: Pulsed electromagnetic field ameliorates the progression of osteoarthritis via the Sirt1/NF-κB pathway
Source: Arthritis Res Ther. 2025 Feb 14;27:33. doi: 10.1186/s13075-025-03492-0 (PMC11827477; doi:10.1186/s13075-025-03492-0)
Supplement: Supplementary file 1 — Supplementary Material 1 [file 13075_2025_3492_MOESM1_ESM.pdf]

Raw images of Western blot in Figure. 2.

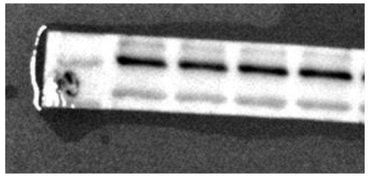

GAPDH

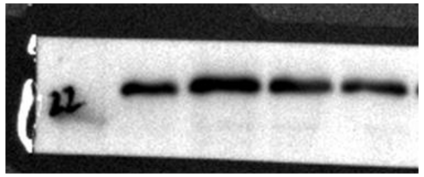

Cox-2

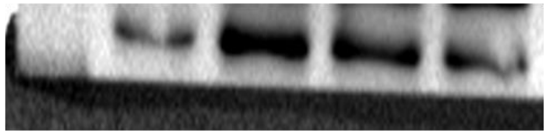

iNOS

Raw images of Western blot in Figure. 4.

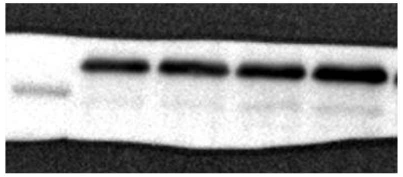

Lamin B

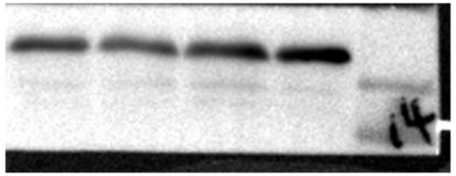

Sirt1

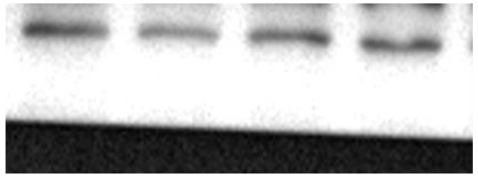

IκBα

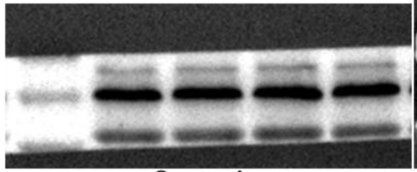

β-actin

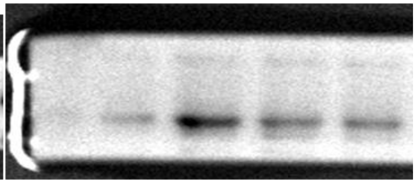

P65 in nucleus

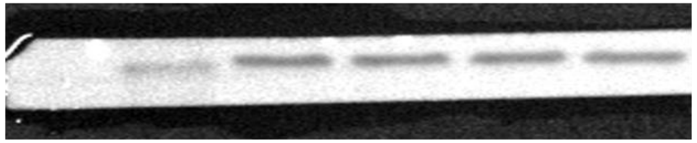

P65 in whole cell

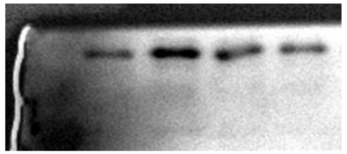

P-P65 in whole cell

Raw images of Western blot in Figure. 5.1.

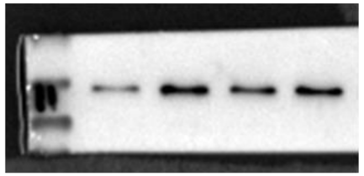

P65

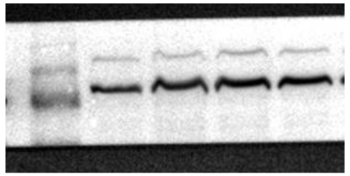

Lamin B

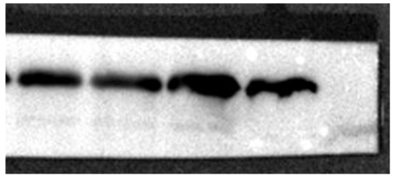

Sirt1

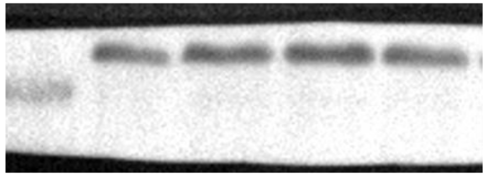

GAPDH

Raw images of Western blot in Figure. 5.2.

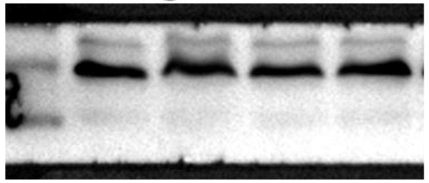

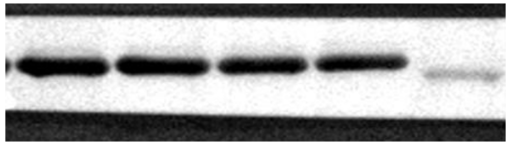

GAPDH
